# Native-valve *Enterococcus hirae* endocarditis: a case report and review of the literature

**DOI:** 10.1186/s12879-019-4532-z

**Published:** 2019-10-24

**Authors:** Mary E. Pinkes, Catherine White, Cynthia S. Wong

**Affiliations:** 1 College of Medicine, SUNY Upstate Medical University, 766 Irving Avenue, Syracuse, NY 13210 USA; 2 Department of Medicine, SUNY Upstate Medical University, University Hospital, 750 E. Adams Street, Syracuse, NY 13210 USA; 3 Department of Infectious Disease, St. Joseph’s Health Center, 301 Prospect Avenue, Syracuse, NY 13203 USA

**Keywords:** *Enterococcus hirae*, Enterococci, Endocarditis, Aortic valve endocarditis

## Abstract

**Background:**

*Enterococcus hirae* is rarely identified in humans and may be a commensal pathogen in psittacine birds. We present the fifth known case of *E. hirae* endocarditis.

**Case presentation:**

A 64-year-old Caucasian female presented with fever, hypotension, atrial fibrillation with rapid ventricular response, and a two-week history of lightheadedness. Her previous medical history included COPD, recurrent DVT, atrial fibrillation (on warfarin), hypertension, hypothyroidism, and Hodgkin’s lymphoma. Physical exam was notable for expiratory wheezes and a 2/6 systolic ejection murmur at the right sternal border. 2D echocardiogram revealed severe aortic stenosis. The patient underwent right and left heart catheterization, where she was found to have severe aortic stenosis and mild pulmonary hypertension. She subsequently underwent minimally invasive aortic valve replacement with a bovine pericardial valve, bilateral atrial cryoablation, and clipping of the left atrial appendage. Her aortic valve was found to have a bicuspid, thickened appearance with calcifications, multiple small vegetations, and a root abscess beneath the right coronary cusp. With a new suspicion of infective endocarditis, the patient was placed on broad-spectrum IV antibiotics. Intra-operative blood cultures were negative. A tissue culture from the aortic valve vegetations identified *Enterococcus hirae* susceptible to ampicillin through MALDI-TOF. Antibiotic treatment was then switched to IV ampicillin and ceftriaxone; she declined aminoglycoside treatment due to toxicity concerns. The patient had an uncomplicated postoperative course and was discharged with 6 weeks of antibiotics. To date, she continues to be followed with no signs of relapsing disease.

**Conclusions:**

To our knowledge, this case constitutes the fifth known case of *E. hirae* endocarditis, and the second case to have been identified with MALDI-TOF and treated with ampicillin and ceftriaxone. This case reinforces the efficacy of ampicillin and ceftriaxone for the treatment of *E. hirae* endocarditis.

## Background

Enterococci are Gram-positive, facultative anaerobes frequently found in the intestinal flora of humans and animals [1]. In recent years, they have attracted notice as an increasingly common source of hospital-acquired infections, particularly with concerns about antibiotic resistance [2]. *Enterococcus faecalis* and *E. faecium* are the most commonly identified species, classically accounting for roughly 80 and 10%, respectively, of all enterococcal infections [1–3]. More recently, these statistics have approached approximately 97% (*E. faecalis*) and 1–2% (*E. faecium*), with about 1% of the remaining enterococcal infections originating from other enterococcal species [4]. *E. hirae* is rarely identified in humans and may be a commensal pathogen in psittacine birds [5]. It has also been associated with poultry and suckling animals [5, 6]. Data on the prevalence of *E. hirae* infections in humans is limited, but *E. hirae* may account for between 0.4 and 3.03% of all enterococcal infections and may be underdiagnosed due to rarity [2, 3]. To our knowledge, this case constitutes the fifth known case of *E. hirae* endocarditis. We review the clinical aspects of the previous four known cases of *E. hirae* endocarditis.

## Case presentation

**Fig. 1:**
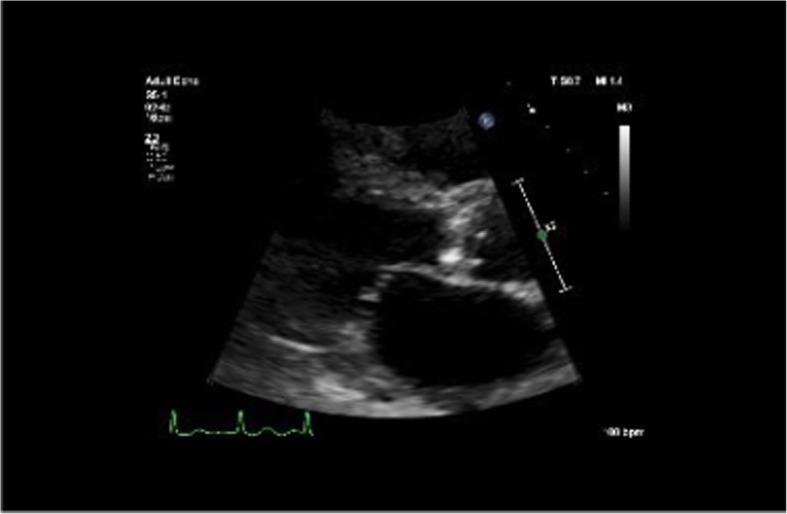
Parasternal long-axis view on 2D transthoracic echocardiogram upon patient's initial presentation, demonstrating normal left ventricular systolic function and severe aortic stenosis with no indications of vegetations or infectious processes

**Fig. 2:**
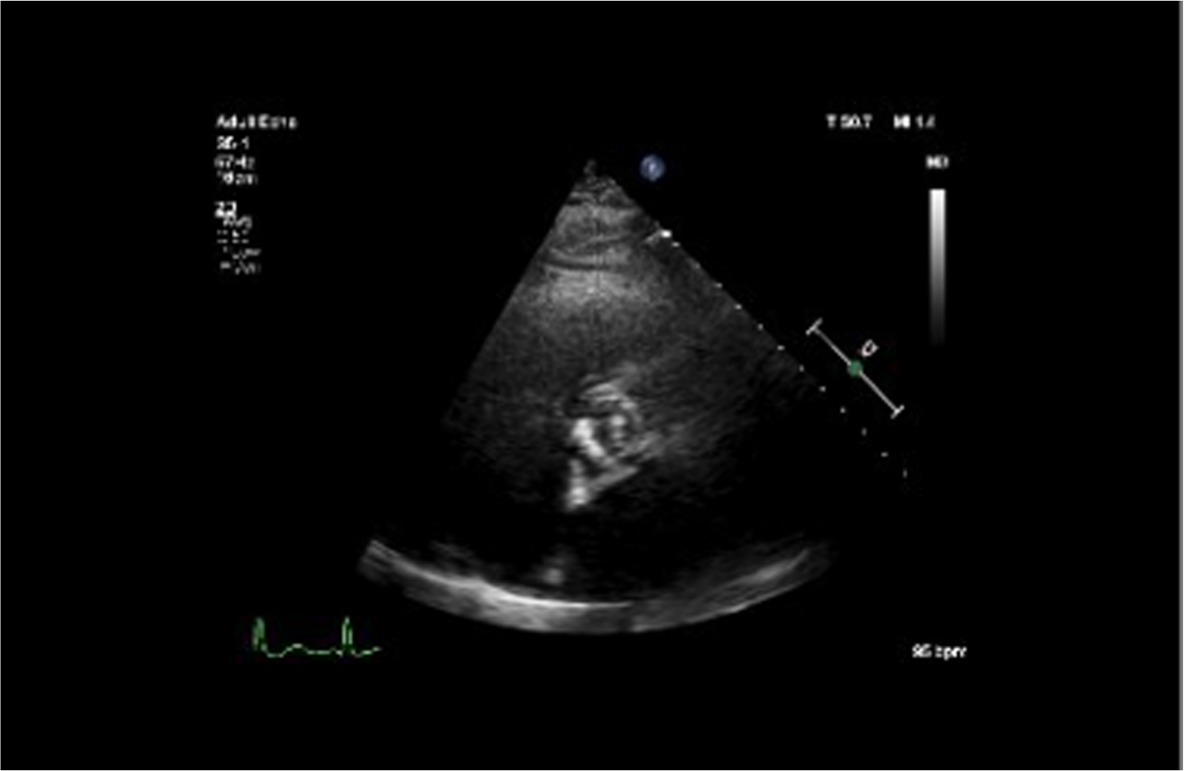
Parasternal short-axis view from the same echocardiogram

A 64-year-old Caucasian female was transferred to a tertiary care hospital with a fever, hypotension, and a two-week history of lightheadedness and dizziness with near-syncopal episodes and mild visual disturbances. She reported a weight loss of 40 pounds over the past 2 years due to progressive dysphagia, and upper endoscopy at the previous hospital revealed esophageal candidiasis. Her previous medical history included COPD, asthma, recurring right lower extremity DVT, achalasia, atrial fibrillation (on warfarin), fibromyalgia, hypertension, hypothyroidism, cholecystectomy, and a distant history of Hodgkin’s lymphoma (for which she received chemotherapy). BMI was 30.62 kg/m^2^. She denied chest pain and shortness of breath. Upon arrival at the hospital, she was found to be in atrial fibrillation with rapid ventricular response and complained of palpitations. Physical exam was notable for expiratory wheezes and a 2/6 systolic ejection murmur at the right sternal border. She spontaneously converted to sinus rhythm after receiving intravenous metoprolol, and her EKG showed T-wave inversion in the inferior leads. Her labs showed a white blood cell count of 8.0 × 10^3^/mm^3^, an INR of 5.7, a hematocrit of 25.6%, a troponin I level of 0.05 ng/mL, and a BNP of 6696 pg/mL. Stool guaiacs were positive. A chest x-ray and CT of the abdomen and pelvis were negative for acute disease. A 2D echocardiogram revealed severe aortic stenosis with a peak gradient of 91 mmHg, a mean gradient of 61 mmHg, and normal systolic left ventricular function (see Figs. 1 and 2). She was given two units of packed red blood cells, four units of fresh frozen plasma, and fluconazole.

Four days after initial presentation, the patient underwent right and left heart catheterization for evaluation of her coronary arteries and aortic valve. She was found to have severe aortic stenosis (mean gradient of 56 mmHg, valve area of 0.64 cm^2^), mild pulmonary hypertension (28 mmHg), and normal left systolic function, with no evidence of significant coronary artery disease. The aortic root size was noted to be normal. Valve surgery was recommended.

The patient underwent minimally invasive aortic valve replacement with a bovine pericardial valve, cryoablation of both atria, and clipping of the left atrial appendage. The aortic valve was found to have a bicuspid, thickened appearance with calcifications, multiple small vegetations, and a root abscess beneath the right coronary cusp. With a new suspicion of infective endocarditis, the patient was placed on broad-spectrum intravenous antibiotics with vancomycin and piperacillin/tazobactam. Two intra-operative peripheral blood cultures were taken, and an infectious disease consult was requested.

One day post-surgery, the level of C-reactive protein was measured at 8.4 mg/L, and the sedimentation rate was 46 mm/hr. Three days post-surgery, a tissue culture from the aortic valve vegetations identified *Enterococcus hirae* through MALDI-TOF. The tissue sample was sensitive to ampicillin, amoxicillin, and vancomycin, and it demonstrated synergy with gentamicin and streptomycin. Antibiotic treatment was then switched to intravenous ampicillin and ceftriaxone; the patient declined aminoglycoside treatment due to concerns of nephrotoxicity. Both blood cultures were negative for bacterial growth. The patient denied any exposure to psittacine birds, poultry, and suckling animals, as well as recent travel.

The patient had an uncomplicated postoperative course. She was discharged with the goal of 6 weeks of treatment with intravenous ampicillin and ceftriaxone.

Two months after her discharge, two peripheral blood cultures were negative for bacterial growth. Ten months after her discharge, the patient presented for multifocal pneumonia, and blood cultures were found to be negative then as well. Fifteen months after her initial presentation, the patient had a follow-up appointment with Cardiology. Mild mitral and mild tricuspid regurgitation were noted, and a 1/6 systolic murmur was appreciated at the right upper sternal border. A comprehensive 2D transthoracic Doppler echocardiogram at the time showed a well seated aortic bioprosthetic valve with normal-sized aortic root and without evidence of dehiscence, paravalvular leak, or aortic stenosis.

## Discussion and conclusions

Though enterococci are estimated to cause 5–15% of bacterial endocarditis, enterococcal endocarditis is a relatively uncommon sequela of enterococcal bacteremia [1]. Between 0.5–8% of patients with enterococcal bacteremia may be diagnosed with enterococcal endocarditis [1, 3, 7]. Risk factors for enterococcal bacteremia and endocarditis have been identified as increased age, male gender, underlying cardiac pathology, and a genitourinary or biliary source [1, 3, 4, 7]. Other factors common to enterococcal endocarditis include aortic or mitral valve involvement and a subacute presentation [1, 7]. In those who abuse drugs, right-sided enterococcal endocarditis appears to be rare; mitral and aortic valve enterococcal involvement remain more common in this population [7].

The five known cases of *E. hirae* endocarditis, as summarized in Table 1, occurred in a 72-year-old male [8], 78-year-old female [9], 56-year-old male [10], 70-year-old male [11], and 64-year-old female. All five cases involved the aortic valve, with one case that involved both the aortic and mitral valves [10]. Additionally, there were underlying risk factors for cardiac pathology in each of the first three cases, as well as in ours: a history of coronary artery disease and percutaneous transluminal coronary angioplasty [8]; prior aortic valve replacement with a bioprosthetic valve [9]; cardiac arrhythmia with prior surgical ablation, and a patent foramen ovale [10]; and a bicuspid aortic valve, respectively. The past medical history for the fourth case was not reported, although it was noted that this patient had lived in Vietnam for 2 years, with recent travel throughout Southeast Asia [11].

**Table 1:** Clinical characteristics of patients with endocarditis caused by *Enterococcus hirae*

Patient	1	2	3	4	5
Age, y	72	78	56	70	64
Sex	M	F	M	M	F
Valve involvement	Aortic insufficiency.	Vegetation on aortic prosthetic valve present during relapse; normal TTE/TEE upon initial presentation.	Aortic and mitral valve reflux.	Severe aortic regurgitation with prolapse, fusion, small echodensity, and perforation of aortic cusps.	Bicuspid aortic valve with stenosis, root abscess, calcifications; no evidence of vegetations or abscess on TTE.
Medical history	Coronary artery disease, percutaneous transluminal coronary angioplasty.	Diabetes mellitus, hypertension, prior aortic valve replacement with bioprosthetic.	Hypertension, diabetes mellitus, cardiac arrhythmia with prior surgical ablation, surgical removal of gastric leiomyoma.	Unknown.	Hodgkin’s lymphoma, asthma, achalasia, recurrent right lower extremity DVT, fibromyalgia, cholecystectomy.
Identification	*sodA*_int_ gene sequencing.	16S rNA, *sodA*_int_ gene sequencing.	Unknown.	MALDI-TOF.	MALDI-TOF.
Treatment	Aortic valve replacement. Ampicillin and gentamicin (4 weeks), with rifampin added (15 days); total 4 weeks.	Amoxicillin and gentamicin (2 weeks), followed by amoxicillin and rifampin (4 weeks); total 6 weeks.	Aortic valve replacement. Ampicillin and gentamicin (4 weeks), followed by amoxicillin and rifampin (2 weeks); total 6 weeks.	Aortic valve replacement. Ampicillin and ceftriaxone, followed by IV penicillin G and ceftriaxone for 6 weeks, with indefinite chronic suppressive therapy of oral penicillin.	Aortic valve replacement. Ampicillin and ceftriaxone (6 weeks).
Relapse	Yes, 3 months after antibiotic discontinuation. Treated with aortic valve replacement and vancomycin/gentamicin (6 weeks) followed by amoxicillin (2 weeks) for a total of 8 weeks of antibiotic therapy. Resolved.	Yes, 4 months after antibiotic discontinuation. Treated with the same regimen as before. Surgery contraindicated by poor patient condition. Resolved.	No.	No.	No.
Notes	Subacute presentation.	Subacute presentation. No evidence of endocarditis on initial TTE/TEE. Multiple colonic polyps removed. Post-relapse, colonic polyp with non-neoplastic adenoma removed. The only case of the five not to require aortic valve replacement, although the infected valve was already a prosthetic valve.	Presented acutely as neurological deficit with slurred speech and left hemiparesis.	Subacute presentation with 3 months of bilateral lower extremity edema, exertional dyspnea, and fatigue; 1 year of fevers and mild weight loss. Lived in Vietnam for 2 years, with travel throughout Southeast Asia.	Presented acutely as hypotension and atrial fibrillation with rapid ventricular response.
Year of publication, reference	2002 [8]	2011 [9]	2013 [10]	2019 [11]	2019

All reported cases of *E. hirae* endocarditis have involved the aortic valve. Presentations of *E. hirae* endocarditis have been both acute and subacute. Treatment requires several weeks of bactericidal treatment, classically ampicillin and gentamicin in combination, and often necessitates aortic valve replacement

*TTE* Transthoracic echocardiography, *TEE* Transesophageal echocardiography

All five cases involved a history of at least 1 month of subacute symptoms, typically fever, weight loss, fatigue, and weakness [8–11]. The fourth case presented with 3 months of bilateral lower extremity edema, exertional dyspnea, and fatigue [11]. Two patients presented acutely, and both acute presentations did not at first appear to be infective endocarditis. One case presented acutely with dysarthria, left hemiparesis, and a brain lesion on MRI [10]. Our case presented with hypotension and atrial fibrillation with rapid ventricular response, in the context of a history of 2 weeks of lightheadedness, dizziness, near-syncopal episodes, and mild visual disturbances.

None of the five cases involved a documented source of entry, including a genitourinary or biliary source, though some patients had distant histories of gastric surgery. Our case had positive stool guaiacs and a remote history of cholecystectomy. Only one case was theorized to have a probable source, in which the patient had had multiple colonic polyps removed and was later found, post-relapse and after a second colonoscopy, to have a non-neoplastic adenoma in a removed polyp [9]. Another patient had a history of gastric leiomyoma removal [10].

Previous cases have discussed the need for definitive genetic species identification of *E. hirae*. In the first two cases, the API 20 Strep, rapid ID 32 Strep, and IDGP N052 card systems could not accurately identify *E. hirae*, and definitive identification with *sodA*_int_ and/or 16S rRNA gene sequencing was required [8, 9]. There has also been an example of *Lactococcus garvieae* that was misidentified by the Vitek2® automated system (bioMérieux, Marcy l’Étoile, France) as *E. hirae* [12]. The fourth case was identified using MALDI-TOF [11], as was ours.

All five cases responded to antibiotic treatment, and all five patients survived. The first three cases were treated with ampicillin or amoxicillin, plus gentamicin, followed by rifampin. Four of the five cases, including ours, resulted in aortic valve replacement [8, 10]. In the only case that did not require a valve replacement, the infected aortic valve was already a prosthetic valve, and the patient’s poor condition contraindicated surgery [9]. Two patients relapsed several months following antibiotic discontinuation [8, 9]. The first of the two relapses resolved after treatment with vancomycin, gentamicin, and amoxicillin, as well as aortic valve replacement [8]. The second relapse, in which the patient already had a prosthetic aortic valve, resolved after repetition of the initial treatment, amoxicillin, gentamicin and rifampin [9]. The fourth case was treated with ampicillin and ceftriaxone, following treatment guideline changes [4], and the patient was discharged on 6 weeks of IV penicillin G and ceftriaxone, followed by indefinite chronic suppressive therapy with oral penicillin [11]. Our case was treated with 6 weeks of ampicillin and ceftriaxone, with no evidence of relapse at 15 months after the patient’s discharge.

Bactericidal antibacterial activity against enterococci has classically required the combination of a β-lactam antibiotic, such as ampicillin, with an aminoglycoside, such as gentamicin. In the population typically affected by enterococcal endocarditis, however, patients tend to be older, to have multiple comorbid conditions, and to have poorer renal function at baseline. For these patients, therefore, the risks of aminoglycoside-associated nephrotoxicity leading to renal failure are increased in a treatment course of several weeks, and may outweigh the benefits of aminoglycoside use [4].

Our case reinforces previous findings that *E. hirae* endocarditis may be successfully treated with ampicillin and ceftriaxone, which may allow patients to avoid the significant toxicities of gentamicin.

## Data Availability

Data sharing is not applicable to this article as no datasets were generated or analyzed during the current study.
